# Toward Understanding the Molecular Role of SNX27/Retromer in Human Health and Disease

**DOI:** 10.3389/fcell.2021.642378

**Published:** 2021-04-15

**Authors:** Mintu Chandra, Amy K. Kendall, Lauren P. Jackson

**Affiliations:** ^1^Department of Biological Sciences, Vanderbilt University, Nashville, TN, United States; ^2^Center for Structural Biology, Vanderbilt University, Nashville, TN, United States; ^3^Department of Biochemistry, Vanderbilt University, Nashville, TN, United States

**Keywords:** membrane traffic, retromer complex, sorting nexin 27, structural biology, coat proteins

## Abstract

Aberrations in membrane trafficking pathways have profound effects in cellular dynamics of cellular sorting processes and can drive severe physiological outcomes. Sorting nexin 27 (SNX27) is a metazoan-specific sorting nexin protein from the PX-FERM domain family and is required for endosomal recycling of many important transmembrane receptors. Multiple studies have shown SNX27-mediated recycling requires association with retromer, one of the best-known regulators of endosomal trafficking. SNX27/retromer downregulation is strongly linked to Down’s Syndrome (DS) via glutamate receptor dysfunction and to Alzheimer’s Disease (AD) through increased intracellular production of amyloid peptides from amyloid precursor protein (APP) breakdown. SNX27 is further linked to addiction via its role in potassium channel trafficking, and its over-expression is linked to tumorigenesis, cancer progression, and metastasis. Thus, the correct sorting of multiple receptors by SNX27/retromer is vital for normal cellular function to prevent human diseases. The role of SNX27 in regulating cargo recycling from endosomes to the cell surface is firmly established, but how SNX27 assembles with retromer to generate tubulovesicular carriers remains elusive. Whether SNX27/retromer may be a putative therapeutic target to prevent neurodegenerative disease is now an emerging area of study. This review will provide an update on our molecular understanding of endosomal trafficking events mediated by the SNX27/retromer complex on endosomes.

## Introduction

Cells communicate with the extracellular environment via cell surface transmembrane proteins that direct processes such as nutrient uptake, cellular adhesion, and intracellular signal transduction. Homeostasis of these molecules is precisely controlled by balancing exocytic, endocytic, and intracellular trafficking pathways. How these pathways are connected and regulated is a question of fundamental and immense interest, both for understanding normal cell physiology and the etiology of important human diseases. Mechanisms that regulate transmembrane cargo sorting within endosomes remain poorly understood. In neurons, proteins and lipids must be exchanged and remodeled at the cell surface to maintain synaptic plasticity and cognitive development (Anggono and Huganir, 2012). Many critical transmembrane proteins and lipids must be internalized, while others undergo selective sorting, either through recycling to the cell surface or trafficking to lysosomes for down-regulation or degradation. The endocytic network regulates both structural and functional synaptic remodeling by controlling the trafficking of numerous transmembrane proteins cargoes; examples include cell adhesion molecules, receptors required in signaling pathways, and ion channels (Anggono and Huganir, 2012; Di Fiore and von Zastrow, 2014).

**FIGURE 1:**
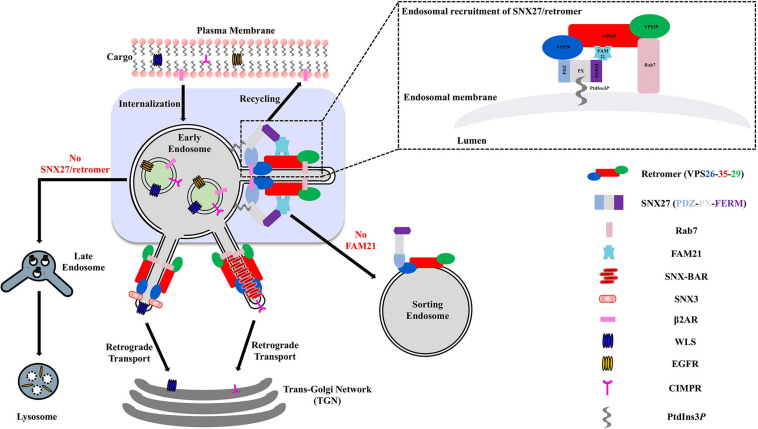
Overview of SNX27/retromer pathway in metazoan cells. Metazoan retromer is implicated in three distinct endosomal pathways through direct interactions with SNX proteins to form elongated tubules. The SNX27/retromer pathway is specific to metazoans and mediates cargo recycling from endosomes to the plasma membrane. In this pathway, cargoes including β2 adrenergic and glutamate receptors contain PDZ binding motifs recognized by SNX27. In addition, the SNX27 FERM domain binds NPxY motifs found in transmembrane cargoes. SNX-BAR/retromer and SNX3/retromer pathways occur in both yeast and metazoans. SNX-BAR/retromer retrieves cargoes from endosomes to the TGN, while SNX3/retromer is implicated in sorting receptors like Wntless (WLS) from endosomes to the TGN.

In metazoans, the retromer complex is considered a “master regulator” of protein sorting at endosomal membranes. The retromer heterotrimer is formed from Vacuolar Protein Sorting protein 35 (VPS35), VPS29, and VPS26 (Seaman et al., 1998; Kerr et al., 2005; Burd and Cullen, 2014). Retromer sorts transmembrane proteins away from degradation in lysosomes and instead sorts proteins back to the cell surface; to the *trans*-Golgi network (TGN); or to specialized endosomes (Figure 1; Seaman et al., 1998; Arighi et al., 2004; Carlton et al., 2004; Seaman, 2004; Chen et al., 2010; Temkin et al., 2011). The functional retromer complex is formed through binding members of the Phox homology (PX) protein family. Retromer binds multiple PX proteins from the sorting nexin (SNX) family, including dimers of SNX1/SNX2 with SNX5/SNX6 (Cullen and Korswagen, 2011; Seaman, 2012; Burd and Cullen, 2014; Simonetti et al., 2017; Chandra et al., 2020); monomeric SNX3 (Xu et al., 2001; Hierro et al., 2007; Strochlic et al., 2007; Harterink et al., 2011; Seaman, 2012; Burd and Cullen, 2014; Lucas et al., 2016; Leneva et al., 2020); and its most recently identified partner, sorting nexin 27 (SNX27) (Gallon et al., 2014; Clairfeuille et al., 2016; Chandra et al., 2020). Membrane remodeling can occur when retromer binds SNX-BAR proteins (SNX1/SNX2, SNX5/SNX6) that induce the formation of tubular transport carriers (Wassmer et al., 2007, 2009; Temkin et al., 2011; van Weering et al., 2012a, b). Very recently, SNX3/retromer has also been shown to induce tubulation *in vitro* (Leneva et al., 2020) in the absence of BAR domains, suggesting BARs are not required for membrane tubulation in the context of retromer coats.

Retromer is thought to coordinate cargo sorting in two ways: by selecting cargo based on specific sequences and by promoting membrane remodeling to form tubular carriers enriched in certain cargoes (Cullen and Korswagen, 2011; Seaman et al., 2013; Burd and Cullen, 2014). Cargo recognition and binding use at least two mechanisms. Some cargoes directly bind VPS35 and VPS26 subunits (Seaman, 2007; Tabuchi et al., 2010; Fjorback et al., 2012). Increasing evidence suggests retromer uses various SNX proteins as cargo adaptors (Strochlic et al., 2007): SNX3 (Xu et al., 2001; Strochlic et al., 2007; Burd and Cullen, 2014), SNX5 (Simonetti et al., 2017, 2019), and SNX27 (Temkin et al., 2011) are all implicated in cargo recognition. Mutations in or loss of functional retromer have been increasingly linked with neurological disorders (Willnow and Andersen, 2013; Reitz, 2015). In this review, we provide an update on the current understanding of SNX27/retromer biology with focus on molecular details and the link between SNX27 and retromer in sorting critical cargoes required for human health.

## SNX27 and Retromer in Endosomal Trafficking

### Identification of Retromer in Eukaryotes

Genetic screening in *Saccharomyces cerevisiae* identified more than 40 “Vacuolar protein sorting” (Vps) genes required for the efficient lysosomal trafficking of acid hydrolases (Bankaitis et al., 1986; Rothman and Stevens, 1986; Robinson et al., 1988; Rothman et al., 1989). Subsequently, several Vps proteins (Vps29, Vps26, Vps35, Vps5, and Vps17) were shown to form a multiprotein complex to transport the transmembrane protein hydrolase receptor Vps10 in a retrograde direction from endosomes to the Golgi (Horazdovsky et al., 1997; Nothwehr and Hindes, 1997; Seaman et al., 1997, 1998). Later, the pentameric complex containing Vps29, Vps26, Vps35, Vps5, and Vps17 proteins was named “retromer” (Seaman et al., 1998). Budding yeast retromer is regarded to exist in a stable pentameric complex (Seaman, 2012; Burd and Cullen, 2014) composed of two subcomplexes: the Vps35/Vps26/Vps29 heterotrimer and Vps5/Vps17 heterodimer (Horazdovsky et al., 1997; Seaman et al., 1998).

In metazoans, recent evidence suggests the retromer heterotrimer has diverged functionally from its role in yeast (Seaman, 2012; Burd and Cullen, 2014). The mammalian homologs of the Vps35/Vps26/Vps29 heterotrimer are VPS35, VPS26A/VPS26B, and VPS29, respectively (Haft et al., 2000; Koumandou et al., 2011); in this review, we refer to this heterotrimer as “retromer.” Differential incorporation of VPS26A or VPS26B subunits into retromer likely allows differential cargo sorting abilities (Bugarcic et al., 2011; McMillan et al., 2016). Functionally, SNX1 and SNX2 are considered equivalents of yeast Vps5, while SNX5 and SNX6 are Vps17 equivalents (Horazdovsky et al., 1997; Carlton et al., 2004; Seaman, 2004; Wassmer et al., 2007, 2009; Koumandou et al., 2011). These metazoan proteins likely arose from gene duplication. The SNX1/2:SNX5/6 heterodimer is the mammalian counterpart of yeast Vps5/Vps17, and it contains both PX and Bin/Amphiphysin/Rvs (BAR) domains (Cullen, 2008). The SNX-BAR heterodimer has long been considered responsible for membrane remodeling to promote cargo recycling and has been referred to as the “membrane deformation complex” (van Weering et al., 2010; Burd and Cullen, 2014). More recently, the SNX-BAR dimer has been shown to bind and sort cargo in a retromer-independent manner as the ESCPE-1 complex [(Simonetti et al., 2017, 2019; Evans et al., 2020); recently reviewed in Chandra et al. (2020)].

### Retromer-Mediated Cargo Recognition

Following identification as a multiprotein trafficking complex in budding yeast, mammalian retromer has been implicated in sorting hundreds of transmembrane cargoes either to the TGN or to the cell surface by re-routing away from degradation in lysosomes (Cullen and Steinberg, 2018). Retromer recycles many important transmembrane cargoes from the endosome to the TGN, including sortilin (Mari et al., 2008), SorLA (Fjorback et al., 2012), and SorCS1 (Lane et al., 2010). However, in metazoans, retromer was later found to sort hundreds of transmembrane proteins from endosomes to the plasma membrane via an interaction with SNX27, which has become an emerging player implicated in recycling of solute carriers, glutamate receptors, and potassium channels (Lunn et al., 2007; Lauffer et al., 2010; Steinberg et al., 2013; Clairfeuille et al., 2016; Yang et al., 2018). In recent years, the role of retromer in the endosome-to-plasma membrane recycling pathway has emerged as critical to cellular and human health.

### SNX27

**FIGURE 2:**
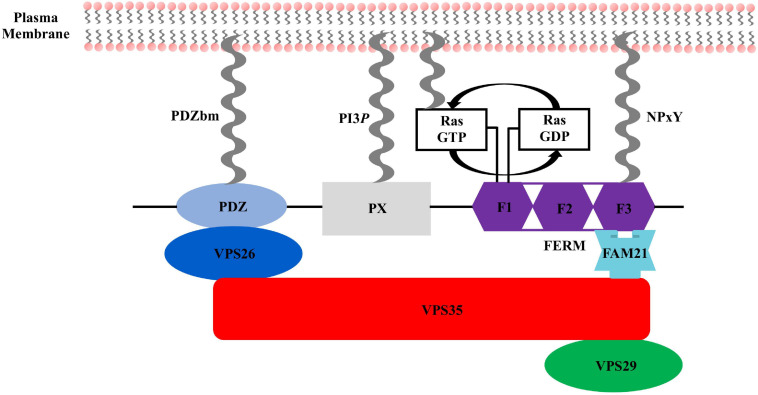
Sorting nexin 27 (SNX27) domain architecture. SNX27 contains a modular architecture with three established domains: the N-terminal PDZ domain (light blue), middle PX domain (gray), and C-terminal FERM domain (purple), which is divided into F1, F2, and F3 sub-domains. Key interactions in the SNX27/retromer pathway are indicated. VPS26 (dark blue) binds the PDZ domain. Ras binds the F1 sub-domain, and FAM21 engages both the SNX27 F3 sub-domain and retromer VPS35 subunit. All three SNX27 domains directly engage protein partners embedded in membranes enriched for PI3*P*.

**FIGURE 3:**
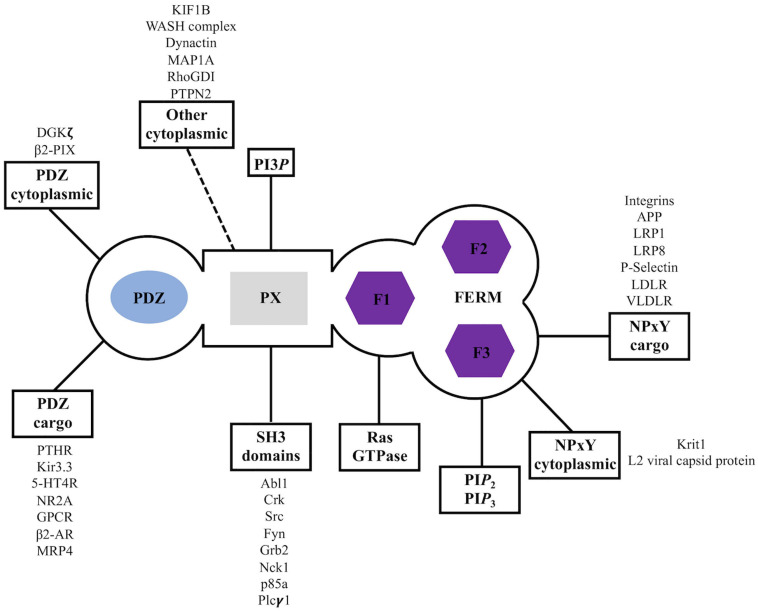
Sorting nexin 27 (SNX27) interaction partners. Summary of reported binding partners for SNX27 structural domains.

The sorting nexin, SNX27, contains an N-terminal post-synaptic density 95/discs large/zonula occludens-1 (PDZ) domain; central PX domain; and C-terminal band 4.1/ezrin/radixin/moesin (FERM) domain (Figure 2). SNX27 is predominantly expressed in brain and was first studied at the protein level using proteomics, which revealed it interacts with the G-protein-coupled receptor (GPCR) called 5-hydroxytryptamine type 4 receptor (5-HT_4__(a)_R) (Joubert et al., 2004). 5-HT_4__(a)_R possesses a class I PDZ binding motif (PDZbm) at its C-terminus with the consensus sequence motif X-S/T–X–Φ, where Φ represents any hydrophobic residue. Truncation studies concluded 5-HT_4__(a)_R associates with SNX27 in a PDZ dependent manner. Subsequent work demonstrated SNX27 recognizes specific receptors containing PDZ motifs via its PDZ domain; these cargoes include GPCRs, ion channels, and neuronal proteins (Lunn et al., 2007; Rincon et al., 2007; Lauffer et al., 2010; Cai et al., 2011; Valdes et al., 2011; Hayashi et al., 2012; Munoz and Slesinger, 2014). SNX27 has now been shown to engage many protein and lipid partners; a summary is shown in Figure 3, and we discuss key partners below.

### SNX27 Membrane and Cargo Binding

**FIGURE 4:**
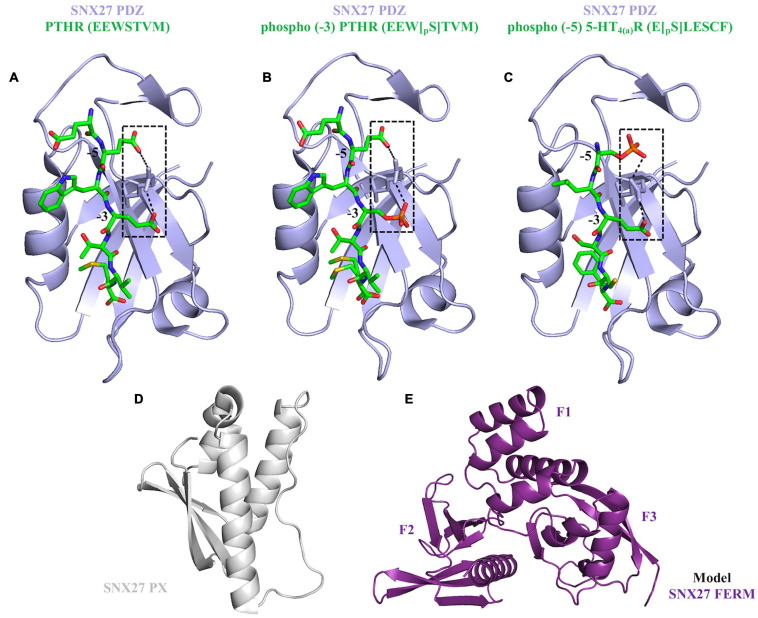
Current structural overview of SNX27. **(A–C)** The SNX27 PDZ domain (purple ribbons) bound to PDZ binding motifs (green cylinders). These motifs can take three different forms: unphosphorylated (PDB ID: 4Z8J) shown in panel **(A)**; phosphorylated at the –3 position (PDB ID: 5EMB) shown in panel **(B)**; and phosphorylated at the –5 position (PDB ID: 5EM9) shown in panel **(C)**. Phosphorylation state is a further way to module binding through increased affinity. **(D)** SNX27 PX domain shown in gray ribbons (PDB ID: 4HAS). **(E)** The SNX27 FERM domain model was generated from the X-ray crystal structure of SNX17 FERM domain bound to the NPxY motif found in P-selectin (PDB ID: 4GXB); the F1, F2, and F3 sub-domains are labeled.

Several studies have shown SNX27 must be directed to endosomes in order to ensure trafficking of protein cargoes (Stockinger et al., 2002; Joubert et al., 2004; van Kerkhof et al., 2005; Czubayko et al., 2006; Lunn et al., 2007; Rincon et al., 2007; Simonetti et al., 2017). SNX27 demonstrates absolute specificity for PI3*P* headgroup association (micromolar binding affinities), and its PX domain drives localization to PI3*P* enriched membranes (Stockinger et al., 2002; Joubert et al., 2004; Knauth et al., 2005; van Kerkhof et al., 2005; Rincon et al., 2007; Lee et al., 2008; Donoso et al., 2009). Structure-based mutagenesis established the dependency of the PX-PI3*P* interaction for membrane recruitment (Misra et al., 2001; Chandra et al., 2019). However, synergistic binding of other modules (PDZ and FERM) to membrane anchored cargo proteins promotes cooperativity for membrane localization (Lauffer et al., 2010; Rincon et al., 2011; Ghai et al., 2013, 2015). This process is referred to as “coincidence detection” and is established as a fundamental physical requirement for the highly specific assembly of transport machineries at different organelles. The SNX27 PDZ domain binds both transmembrane and cytosolic proteins using type-I PDZ binding motifs (consensus sequence: X-S/T–X–Φ, where Φ represents any hydrophobic residue) (Joubert et al., 2004; Lunn et al., 2007; MacNeil et al., 2007; Rincon et al., 2007; Lauffer et al., 2010). Structural studies (Figure 4) have elucidated the molecular basis for PDZbm cargo recognition. The PDZbm sequences are often found in the protein C-terminus, and they possess acidic side chains at the −3 and −5 positions that form an “electrostatic clamp” with a conserved arginine on the SNX27 surface and thereby enhance affinity (Figure 4A; Clairfeuille et al., 2016). Many SNX27 PDZbms, including those found in NMDARs and β2 adrenergic receptor (β_2_AR), lack these upstream acidic side residues; instead, they possess conserved phosphorylation sites on serine and threonine residues (Clairfeuille et al., 2016). Crystal structures of SNX27 PDZ domain bound to different phosphorylated peptides showed how Ser/Thr phosphorylation functions to “mimic” the acidic side chains required for high affinity binding (Figures 4B,C; Clairfeuille et al., 2016).

The FERM domain comprises three sub-domains called F1, F2, and F3 (Figures 2, 4E). This domain has been proposed to regulate interactions with endosomal cargos and/or to serve as a scaffold for signaling complexes (Ghai and Collins, 2011; Ghai et al., 2011). The F1 subdomain contains a predicted ubiquitin-like fold. The F3 subdomain is predicted to have a structure similar to the pleckstrin homology (PH) and phosphotyrosine binding (PTB) domains based on sequence predictions (Stolt et al., 2003, 2005). The F1 and F3 modules are somehow oriented by the central F2 subdomain that contains four α-helices. The sequence identity of SNX27 FERM compared with the “canonical” FERM domain from SNX17 and SNX31 is low, and F2 is much smaller than its equivalents (Ghai et al., 2011). The SNX27 FERM domain can bind Ras GTPases via the F1 module (Ghai and Collins, 2011), while the F3 subdomain binds cargo receptors using short NPxY motifs present in the cytosolic tails of activated signaling receptors (Ghai et al., 2011). The ability of both the PDZ and FERM domains to bind cargo motifs significantly extends the repertoire of potential cargo molecules. SNX27 also binds negatively charged phosphoinositides via the F3 module, which contains a binding site with high affinity for specific phosphoinositide head groups (Mani et al., 2011; Ghai et al., 2015) enriched at the PM and within late endosomal compartments. This suggests a potential mechanism for activation-dependent redistribution of SNX27 to the plasma membrane. The role of SNX27 at the plasma membrane remains uncharacterized, but its recruitment to the contact zone between T cells and the APC (antigen presenting cell) may be important for maintaining the immunological synapse (IS) by controlling endocytic sorting and signaling down-regulation of receptors, such as Disabled homology 1 (Dab1) in reelin signaling (Stolt et al., 2003, 2005; Ghai et al., 2015). Overall, the distinct endosomal and PM localization of SNX27 may be partly explained by the presence of both phosphoinositide and cargo binding modules in its C-terminal FERM domain.

### SNX27/Retromer

**FIGURE 5:**
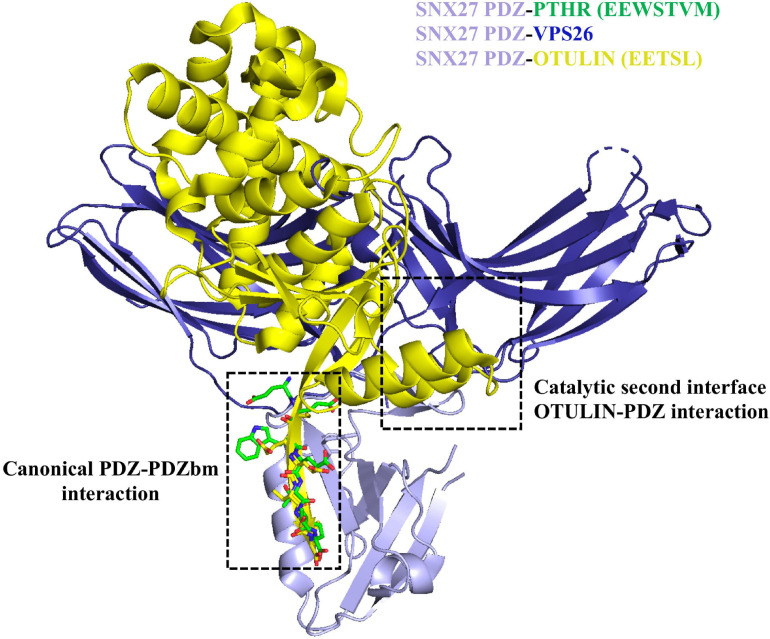
Sorting nexin 27 (SNX27) binding to retromer and OTULIN. The SNX27 PDZ domain (light purple ribbons; PDB: 4Z8J) with a PDZbm (green cylinders) is shown with both VPS26 (dark blue ribbons; PDB ID: 4P2A) and OTULIN (yellow ribbons; PDB ID: 6SAK). Structural data suggest the interaction of SNX27 PDZ with retromer/cargo or OTULIN is mutually exclusive. OTULIN engages both the PDZbm site using a class I PDZbm and the β-hairpin required for interacting with VPS26.

In metazoans, SNX27/retromer has been established as a coat that recycles specific cargoes from endosomes to the plasma membrane. SNX27/retromer cargo recycling is thought to occur on Rab4-positive early endosomes and requires the SNX-BAR complex (Cullen and Korswagen, 2011; Temkin et al., 2011; Steinberg et al., 2013). SNX27 acts as a major trafficking regulator through binding PDZ cargo in mammalian cells (Cao et al., 1999; Lauffer et al., 2010; Temkin et al., 2011; Steinberg et al., 2013; Gallon et al., 2014). In this pathway (Figure 1), SNX27 must first be recruited to endosomes when its the PX domain binds PI3*P*, perhaps with an additional contribution from the FERM domain (Ghai et al., 2015); data indicate disrupting the FERM/PI3*P* interaction reduces SNX27 association with endosomal recycling compartments (Ghai et al., 2015). Structural studies have demonstrated a direct interaction between VPS26 and the SNX27 PDZ domain (Gallon et al., 2014). An X-ray crystal structure revealed an exposed β-hairpin on the PDZ domain that binds a conserved groove on the VPS26 surface (Figure 5). Association between the SNX27 PDZ domain and VPS26 increases affinity for PDZ binding motifs, which hints how cargo sorting may be allosterically regulated by SNX27/retromer complex formation (Gallon et al., 2014).

### SNX27 Binding Partners

Overall, SNX27 has been primarily linked to membrane trafficking of cargo proteins via binding to either PDZbm or NPxY motifs. However, SNX27 has been demonstrated to bind other molecules and perhaps influence their mode of action; understanding these additional protein-protein interactions remains important (Teasdale and Collins, 2012), especially since we lack molecular details for many binding partners.

Another established interaction partner for SNX27 is a protein from the WASH complex called FAM21 (or WASHC2) (Temkin et al., 2011; Freeman et al., 2014; Kvainickas et al., 2017). The WASH complex contains five proteins: WASH1; WASHC2 (FAM21); WASHC3 (formerly KIAA1033); WASHC4 (or strumpellin); and WASHC5 (formerly CCDC53) (Derivery et al., 2009). Unlike retromer, the WASH complex is not conserved across evolution and is absent in multiple organisms including yeast. The WASH complex mediates F-actin filament formation on endosomal membranes and is required for endosome-to-cell surface recycling (Seaman et al., 2013). FAM21 has been reported to prevent retrieval of the glucose transporter, GLUT1, to the Golgi and direct it into SNX27/retromer recycling pathway (Lee et al., 2016). In mammals, WASH complex is recruited to endosomes through a direct interaction between FAM21 and VPS35 (Harbour et al., 2012; Jia et al., 2012; Helfer et al., 2013). The interaction between SNX27 and FAM21 may thus be important in the context of SNX27/retromer coat assembly or regulation, but molecular details describing how SNX27 engages FAM21 are currently unknown.

Sorting nexin 27 also interacts with the monomeric small GTPase, Ras (Ghai and Collins, 2011; Ghai et al., 2011), which is associated with multiple signaling pathways implicated in oncogenic signaling (Herrero et al., 2016). The Ras interaction occurs through the FERM F1 subdomain, which is also implicated in binding NPxY cargo proteins (Burden et al., 2004; Ghai and Collins, 2011; Ghai et al., 2011). These data may suggest other FERM domain proteins possess similar binding activity. Krit1 has been identified as an effector for Rap1, another Ras family protein; Krit1 interacts with Rap1 through its FERM domain and stabilizes epithelial junctions (Serebriiskii et al., 1997; Wohlgemuth et al., 2005; Glading et al., 2007; Francalanci et al., 2009). The GIRK (G-protein regulated inward rectifying potassium) class of potassium channels regulates neuronal excitability, and they also depend on the SNX27 FERM domain for localization and trafficking (Balana et al., 2013). In cells expressing dominant negative Ras, SNX27 cannot effectively regulate cell surface levels of GIRK potassium channels (Balana et al., 2013), which suggests a link between Ras regulation and cargo sorting by SNX27.

Finally, the deubiquitinating enzyme (DUB) called OTULIN has recently been shown to interact with SNX27 (Stangl et al., 2019). OTULIN specifically hydrolyzes Met1-linked ubiquitin chains. OTULIN binds two separate surfaces on the SNX27 PDZ domain (Figure 5) with high affinity; it is thought to compete non-catalytically for cargo and retromer binding (Stangl et al., 2019). OTULIN contains a conserved class I PDZbm (sequence: ETSL) essential for binding SNX27. An X-ray crystal structure of OTULIN-SNX27 PDZ revealed a second interface in addition to the canonical PDZ-PDZbm interaction. In the second interface, part of the OTULIN catalytic domain (residues 67–79 containing an exposed β3–β4 hairpin loop) is located in close proximity to the SNX27 PDZ domain, which also engages VPS26A. Compared to the PDZbm alone, OTULIN catalytic domain affinity for SNX27 PDZ is increased over 30-fold (∼30 nM). This represents the tightest interaction ever reported for a PDZ interactor. It appears SNX27 cannot undertake simultaneous binding to both VPS26A and OTULIN (Figure 5), because OTULIN and VPS26A use a partially overlapping binding site located in the SNX27 β3–β4 hairpin loop and would experience clashes between atoms. The existence of other secondary interfaces could modulate the affinity, and thus the selectivity, of SNX27 PDZ interactors; it will be interesting to see if others are identified in future work.

### Open Questions in Cell Biology

Sorting nexin 27 has been linked to endosomal trafficking through a direct interaction with retromer. There is good evidence for describing SNX27 as a retromer “cargo adaptor.” But multiple important questions remain, and further work must be undertaken to understand functional links between the SNX27/retromer complex and Ras. For instance, does SNX27 associate with Ras, retromer, and/or WASH simultaneously? Biochemical experiments and biophysical assays could address this question in the context of artificial membranes to more closely mimic cellular conditions. Is SNX27 endosomal cargo binding affected or regulated by its interaction with Ras? Or might SNX27 somehow regulate Ras function in different signaling pathways? Overall, the ability of SNX27 to associate with and sort transmembrane receptors, and its potential for interacting with small GTPases on endosomal membranes, implicates SNX27 as a potential hub where both endosomal trafficking and integrated signaling processes meet.

It is also important to note how other evidence suggests SNX27 can operate at least somewhat independently of retromer. For example, siRNA-mediated knockdown of SNX27 does not seem to affect VPS35 steady state protein levels, and vice versa. Knockdown experiments indicate SNX27 and retromer only partially overlap in their cargo repertoires (Cullen and Korswagen, 2011; Simonetti et al., 2017; Yong et al., 2020), which further suggests SNX27 can function independently of retromer. Finally, data have suggested a role for SNX27 in internalization from the cell surface, as opposed to the recycling role revealed by studies on β_2_AR (Lunn et al., 2007; Lauffer et al., 2010; Temkin et al., 2011; Hayashi et al., 2012). The role of SNX27 in the intracellular transport of GPCRs, ion channels, and kinases, suggests a possible role in attenuation or propagation of signal transduction, but how SNX27 fulfills both roles remains an important open question.

## Toward a Molecular Understanding of SNX27/Retromer

### SNX27 Structural Studies

The molecular basis of SNX27 binding to PDZ motifs has been established using X-ray crystallography (Figures 4A–C) and biophysical assays (Clairfeuille et al., 2016). Many important cargoes contain PDZ motifs. Examples include GPCRs such as β_2_AR (Lauffer et al., 2010; Choy et al., 2014; Clairfeuille et al., 2016) and parathyroid hormone receptor (PTHR) (Chan et al., 2016; Clairfeuille et al., 2016); ion channels (Lunn et al., 2007; Balana et al., 2011; Clairfeuille et al., 2016); critical neuronal proteins, including α-amino-3-hydroxy-5-methyl-4-isoxazolepropionic acid receptors (AMPARs) (Hussain et al., 2014; Loo et al., 2014; Clairfeuille et al., 2016), NMDA receptors (NMDARs) (Wang et al., 2013; Clairfeuille et al., 2016), and 5-hydroxytryptamine 4a receptors (5-HT_4__(a)_Rs) (Joubert et al., 2004; Clairfeuille et al., 2016) and others (Steinberg et al., 2013; Clairfeuille et al., 2016; Lee et al., 2016). These PDZbm sequences contain the established C-terminal consensus motif (X-S/T–X–Φ) with specific acidic side chains known to interact with a conserved arginine on SNX27 to enhance affinity (Figure 4A). Ser or Thr phosphorylation at the −3 or −5 positions further enhances PDZ motif binding by SNX27 (Clairfeuille et al., 2016; Figures 4B,C) to proteins such as the NMDA receptors.

The SNX27 PDZ domain also directly interacts with the retromer VPS26 subunit (Figure 5; Steinberg et al., 2013; Gallon et al., 2014; Clairfeuille et al., 2016). A β−hairpin located on the SNX27 PDZ domain binds between the two β−sandwich sub−domains of the VPS26 arrestin fold; this binding site is located next to the PDZbm site, but they do not overlap. The interaction with PDZ cargo does not require a “dual recognition surface,” but PDZ cargo binding affinity is enhanced when SNX27 binds retromer. Therefore, cargo sorting can be synergistically coordinated by a specific SNX27/retromer interaction.

Structurally, the SNX27 PX domain (Figure 4D) adopts a globular fold containing three anti-parallel β-strands followed by three α-helices (Seet and Hong, 2006; Chandra and Collins, 2019; Chandra et al., 2019; Li et al., 2019). Sequence alignments of SNX27 PX domain with other PX family members (Seet and Hong, 2006) indicate multiple conserved regions. This includes specific basic residues, as well as the so-called “PPK loop” located between helices α1 and α2, which contains the consensus sequence defined as ΨPxxPxK (Ψ: large aliphatic amino acids V, I, L, and M) (Seet and Hong, 2006; Chandra and Collins, 2019; Chandra et al., 2019). The structure of SNX27 PX domain revealed a shallow and positively charged surface pocket in a location generally considered to be the binding site for negatively charged headgroups (Seet and Hong, 2006; Chandra and Collins, 2019; Chandra et al., 2019; Li et al., 2019), although this structure did not explicitly contain the head group.

In contrast to its N-terminus, we lack experimental structural information about the SNX27 C-terminus. SNX27 belongs to the same PX subfamily as SNX17, and there are two X-ray crystal structures of SNX17 FERM domain bound to NPxY motifs (P-selectin, PDB: 4GXB; and KRIT1, PDB: 4TKN) (Knauth et al., 2005; Francalanci et al., 2009; Ghai and Collins, 2011; Ghai et al., 2013). However, the sequence identity between SNX27 and SNX17 FERM domains is around 25%, so it will be useful to obtain structural information on the SNX27 FERM domain in the presence of its multiple ligands, including NPxY cargo motifs, Ras, and FAM21. Such information would provide key insights into how SNX27 uses its multi-domain architecture to organize a range of binding partners on membranes.

### Retromer Coats

New and emerging structural studies have been invaluable for understanding how retromer assembles into coats on tubules. Recently, multiple new structures of retromer have been published. Thermophilic yeast SNX-BAR/retromer (Kovtun et al., 2018) and both fungal and metazoan SNX3/retromer (Leneva et al., 2020) coats have been reconstituted and visualized using cryo-electron tomography (cryoET). Different oligomers of murine retromer heterotrimer have been observed using single particle cryoEM (Kendall et al., 2020). We will focus discussion here on implications from the newly determined SNX3/retromer structures because a recent review (Chandra et al., 2020) covered other new structures.

Until recently, SNX-BAR proteins were believed to generate the curvature required for tubulation, and there are no reports of retromer alone driving tubulation. SNX-BARs contain amphipathic helices which can insert into lipid bilayers to robustly drive proteins to membranes and to induce membrane curvature (Pylypenko et al., 2007; Bhatia et al., 2009; van Weering et al., 2012b). Tubule extension is promoted by BAR dimerization to form higher-order tubular lattices composed of SNX-BAR complexes (Frost et al., 2008; Mim et al., 2012). The ability of retromer to transport many different cargo proteins has been explained by its ability to bind different adaptors, including sorting nexin proteins that lack membrane-deforming BAR domains; examples include SNX3, SNX12, and SNX27. SNX-BAR/retromer coats exhibit less regularity than do tubules coated with BAR dimers alone (Wassmer et al., 2009; Kovtun et al., 2018; Sun et al., 2020).

Recently, the first structures of mammalian SNX3/retromer coats (Leneva et al., 2020) revealed formation of elongated coated tubules. These data demonstrate SNX-BARs are not required for *in vitro* tubulation. SNX3/retromer coats consist of arch-like units formed by asymmetrical VPS35 homodimers with VPS26 dimers and two SNX3 molecules located at the membrane. SNX3 binds retromer at an interface located between the VPS26 and VPS35 subunits, with its PI3*P* binding pocket facing the membrane. SNX3 is also attached to the membrane using a membrane insertion loop (MIL), which is a feature found in other membrane binding PX domains (Cheever et al., 2006; Seet and Hong, 2006; Chandra and Collins, 2019). The VPS35-mediated arches in SNX3/retromer coats lack two-fold symmetry: one VPS35 monomer appears more curved, and the two VPS35 subunits form an asymmetric dimer interface using electrostatic residues that were proposed and tested in previous structural and biochemical studies (Kendall et al., 2020). Asymmetric assembly of arches in SNX3/retromer coats was proposed to impose directionality and stoichiometry of adaptor binding to coats. These newly observed SNX3/retromer coat structures provide an important foundation for understanding retromer assemblies with other SNX adaptors that lack BAR domains, including SNX27, which may remodel membranes using a similar mechanism. This hints toward a generalized concept for retromer function in which retromer arches form a scaffold that contributes to or helps support membrane bending and can help propagate curvature and tubulation over long distances by oligomerization.

### Open Questions in Structural Biology

Sorting nexin 27 has been well-established as a binding partner for retromer (Steinberg et al., 2013; Gallon et al., 2014). A major outstanding experimental question is whether SNX27 alone or together with retromer is sufficient to generate tubular carriers *in vitro*. It will be interesting to test directly whether SNX27/retromer forms tubules and if or how SNX27 will generate curvature. SNX27 may use its PX domain to orient itself on the membrane, in a manner similar to SNX3 (Leneva et al., 2020). However, SNX3 contains only one structured domain (PX domain), while SNX27 can engage various membrane-embedded ligands through multiple domains. SNX27 may therefore have additional constraints when engaging retromer.

**FIGURE 6:**
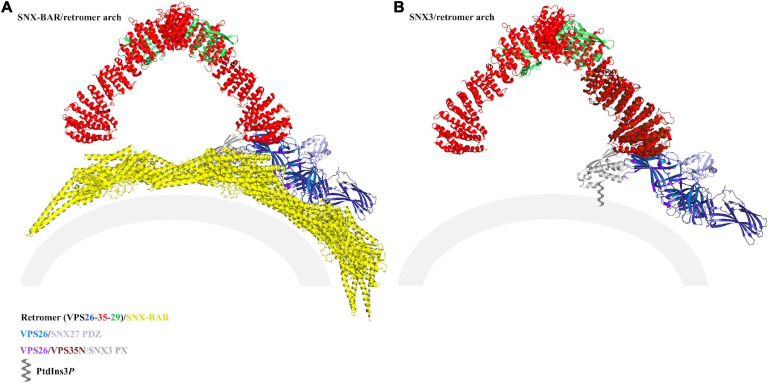
Modeling SNX27/retromer on membranes. Thermophilic yeast SNX-BAR/retromer (PDB ID: 6H7W) **(A)** and mammalian SNX3/retromer coats **(B)** (PDB: not yet available) have been reconstituted *in vitro*. The view in panel **(B)** was generated using PDB: 5F0J, which approximates the reported SNX3/retromer architecture. Both complexes drive tubulation, and reconstructions indicate retromer forms conserved asymmetrical V-shaped arches across eukaryotes. VPS35 is shown as red ribbons, VPS26 as blue ribbons, VPS29 as green ribbons, Vps5 as yellow ribbons, and the SNX3 PX domain as gray ribbons. On each model, potential locations for SNX27 domains are marked. In the SNX-BAR/retromer model **(A)**, the SNX27 PX domain (gray ribbons) appears to be occluded by BAR dimers, and the PDZ domain would likely be blocked from engaging membrane cargo by the BAR layer. In the SNX3/retromer model **(B)**, the SNX27 PX (gray ribbons) and PDZ (purple ribbons) domains are both located close to the membrane. There are currently no structural data regarding the overall architecture of SNX27, either on its own or as part of a retromer coat, so the location of the FERM domain remains unknown. Both models assume the SNX27 PX occupies a similar location to SNX3 PX.

If SNX27/retromer forms coats, then what might these look like? Modeling the SNX27 PDZ interaction with VPS26 in the context of reconstituted arches provides some hints (Figure 6) and raises important new questions. It does not appear SNX27 could bind assembled SNX-BAR/retromer (Figure 6A): the BAR dimers adjacent to the membrane may block or occlude the PDZ domain, which itself needs to bind cargo motifs embedded in the membrane. However, SNX-BAR/retromer has been functionally linked to formation of SNX27/retromer carriers (Steinberg et al., 2013). Could SNX27/retromer somehow “hand off” cargoes to SNX-BAR/retromer, or otherwise engage with SNX-BAR proteins?

It is possible SNX27/retromer could assemble arches in a manner reminiscent to SNX3/retromer, since the SNX27 PX domain contains conserved residues that would allow it to dip into membranes in a similar manner to SNX3. In this scenario (Figure 6B), the SNX27 PX and PDZ domains appear to be located close enough for their linkers to connect the two domains. However, we currently lack information about location and orientation of the FERM domain, so this remains an open question. Alternatively, metazoan retromer has been shown to form oligomers *in vitro*, including longer and flatter chains (Chandra et al., 2020; Kendall et al., 2020) with different and poorly resolved VPS26 links. It remains possible that SNX27/retromer may form a “flatter” coat (Simonetti and Cullen, 2018); superposition of the VPS26/SNX27 PDZ crystal structure onto flat chains reveals the PDZ could in principle be located near membranes, but this has not been observed in the presence of membranes. Overall, reconstitution of SNX27/retromer on PI3P membranes remains an important biochemical and structural target. Reported reconstituted retromer coats appear to assemble tubules with slightly different diameters (Figure 6; Leneva et al., 2020), and this may reflect experimental conditions rather than reality. Could retromer combine with different SNX adaptors to form tubules having different dimensions? This would provide a way for cells to physically direct or sequester cargoes to different tubular carriers originating at the endosome. It will be interesting to see if retromer retains the arches observed in the presence of both SNX-BARs and SNX3; whether retromer can serve as a more adaptable scaffold with the metazoan-specific SNX27; and whether or how SNX-BAR/retromer may be linked structurally to SNX27/retromer.

## SNX27 and Retromer in Human Health and Disease

### Overview

Sorting nexin 27 has been shown to regulate selective endosomal recycling and alter protein composition of cellular membranes through its interaction with retromer. SNX27 is needed to recycle many cargoes that perform essential cellular functions. Therefore, SNX27 drives or influences numerous important processes required for normal human physiology. Examples include neuronal excitability (Balana et al., 2013); synaptic plasticity (Hussain et al., 2014); neural tube development (Belotti et al., 2013); psychostimulant responses (Fujiyama et al., 2003; Joubert et al., 2004); T-cell activation at the IS (Ghai et al., 2015); drug resistance (Hayashi et al., 2012); virion trafficking (Pim et al., 2015); cell motility (Valdes et al., 2011); ion homeostasis (Singh et al., 2015); Wnt signaling (Sun et al., 2016); and glucose transporter recycling (Shinde and Maddika, 2017).

Mouse models revealed SNX27 is required for postnatal growth and survival (Cai et al., 2011). SNX27^–/–^ embryos are viable and develop during embryonic stages, but they show inhibited postnatal growth, including delayed weight gain, reduced organ size, and early death prior to weaning (Cai et al., 2011). The phenotype may arise from aberrant trafficking of NR2C, an ion channel receptor with a C-terminal PDZ motif that binds SNX27. Cai et al. (2011) report NR2C protein, but not mRNA, levels are higher in SNX27^–/–^ mice, and NR2C is not robustly endocytosed in SNX27-deficient neurons. This provides an important molecular link between SNX27 and a key transmembrane protein cargo needed during development. Disruption of SNX27/retromer-mediated endosomal sorting is linked to multiple debilitating neurodegenerative disorders, including Parkinson’s disease (PD) (Harterink et al., 2011; Gallon et al., 2014), Alzheimer’s disease (AD) (Clairfeuille et al., 2016), and Down’s syndrome (DS) (van Weering et al., 2012a). Finally, identification of small molecules that stabilize retromer expression (Mecozzi et al., 2014; Berman et al., 2015; Muzio et al., 2020) underscores the importance of understanding how retromer complexes undergo assembly and regulation. In this section, we briefly highlight the range of cellular pathways influenced by SNX27/retromer.

### Signaling

Sorting nexin 27/retromer has been shown to recycle important signaling receptors, including β_2_ARs (Lee et al., 2008) after ligand-induced endocytosis. Retromer influences cyclic AMP (cAMP) signaling when it recycles PTHR from the early endosome after it dissociates from β-arrestin; this event switches off the signaling pathway (Seaman, 2012; Teasdale and Collins, 2012). Specifically, PTHR has been shown to bind the SNX27 PDZ domain to ensure its recycling (Donoso et al., 2009). SNX27/retromer also trafficks the interferon receptor 2 (IFNAR2) subunit after its endocytosis, a process in which retromer appears to regulate both JAK/STAT signaling termination and gene transcription (Cullen and Korswagen, 2011). SNX27/retromer reduces RANK (receptor activator of NF-κB) signaling in osteoclasts by trafficking RANK in a retrograde pathway to the Golgi (Mim et al., 2012). A study has reported retromer is involved in nucleotide binding-leucine-rich repeat (NB-LRR)-mediated signaling implicated in autophagy (Sun et al., 2020), but it remains unclear how retromer functions in this pathway and whether SNX27 is also involved.

### Autophagy

Autophagy is the process by which cells degrade damaged organelles, misfolded or damaged proteins, and pathogens by enclosing them in a double membrane-bound structure called autophagosomes; these molecules are then delivered to lysosomes for degradation (Cheever et al., 2006). Autophagy is highly conserved across eukaryotes, and cells require autophagy to cope with stress tolerance and signaling induced by nutrients (Simonetti and Cullen, 2018). The involvement of SNX27/retromer in autophagic processes is emerging; it remains unclear whether its role is indirect and whether it is required for autophagy in certain cells (Belotti et al., 2013). SNX27 knockout cells exhibit impaired mTOR complex 1 (mTORC1) activation, which leads to increased autophagy (Yang et al., 2018). The WASH complex regulates trafficking of an essential protein called Atg9 to forming autophagosomes, where it is reported to undertake lipid scrambling and promote autophagosome formation (Maeda et al., 2020); the VPS35 D620N mutation blocks its transport and inhibits autophagy (Fujiyama et al., 2003). Retromer knockdown in *Drosophila* has been shown to disrupt autophagy, when undigested cytoplasmic and endosomal material builds up in autophagosomes (Pim et al., 2015). Retromer has very recently been indirectly implicated in regulation of mTORC1 (Hesketh et al., 2020). Future research is required to understand how SNX27 and/or retromer function alone or together to influence or regulate both autophagy and nutrient sensing.

### Neurodegeneration

Disruption of the endosomal system, and mutations in genes encoding for proteins that play central roles in endosomal trafficking, contribute to pathologies associated with both AD and Parkinson’s disease (PD) (Vilarino-Guell et al., 2011; Wen et al., 2011; Siegenthaler and Rajendran, 2012; Follett et al., 2014; Wang et al., 2014; Reitz, 2015; Small and Petsko, 2015; Mohan and Mellick, 2017; Zhang et al., 2018; Rahman and Morrison, 2019; Vagnozzi and Pratico, 2019; Arbo et al., 2020). SNX27/retromer maintains homeostasis of cell surface receptors, including AMPA and NMDA receptors (Cai et al., 2011; Anggono and Huganir, 2012; Wang et al., 2013; Choy et al., 2014; Hussain et al., 2014; Clairfeuille et al., 2016), in neurons and thus is essential for normal synaptic communication and brain function. Aberrant overactivation of these receptors leads to neuronal hyperactivity and ultimately to seizures commonly associated with epilepsy. In contrast, neuronal hypoactivity can cause synaptic depression linked to many neurodegenerative diseases like AD and PD, as well as to neuropsychiatric disorders like schizophrenia. Collectively, these diseases have an enormous socio-economic impact. A great deal of evidence now supports a direct link between aberrant endosomal trafficking and neurodegenerative disease onset (Chandra et al., 2020). SNX27/retromer clearly plays a critical role in receptor trafficking during synaptic transmission and neuronal function, and thus these proteins have become attractive putative drug targets for brain disorders. Homozygous deletion of SNX27 leads to epilepsy and psychomotor defects; patients typically die within 2 years of birth (Damseh et al., 2015). Here we will highlight specific SNX27 links to DS and AD, since other recent reviews (Wang et al., 2013, 2014; Small and Petsko, 2015; Chandra et al., 2020) have focused on retromer.

In DS, chromosome 21 trisomy drives overexpression of a negative regulator (miR-155) of SNX27, leading to decreased SNX27 expression. SNX27 loss in turn leads to NMDA and AMPA receptor dysfunction associated with DS. Importantly, mouse models suggest that the synaptic and cognitive phenotypes associated with DS can be rescued through SNX27 overexpression (Wang et al., 2013).

Sorting nexin 27 is linked to amyloid precursor protein (APP) trafficking (Steinberg et al., 2013; Wang et al., 2014; Huang et al., 2016) based on proteomic studies of surface protein levels following siRNA knockdown. The link to APP trafficking may occur through another protein, because no direct interactions have been detected between APP and SNX27 (Steinberg et al., 2013). SNX27 has further been implicated in reducing Aβ generation through interactions with PS1/γ-secretase (Wang et al., 2013, 2014). SorLA/SorL1, an intracellular sorting receptor, interacts with APP, and changes in SorLA expression or function affects the cellular distribution and processing of APP. There are now multiple links between SNX27, retromer, and SorLA/SorL1. The retromer VPS26 subunit has been shown to interact with SorLA *in vivo* (Fjorback et al., 2012), but how retromer regulates APP trafficking and processing remains largely unknown. Biochemically, the SNX27 PDZ domain has been shown to bind SorLA with its cytosolic C-terminal FANSHY motif (Nielsen et al., 2007; Huang et al., 2016; Milne et al., 2019), but no structures have been reported. Down-regulation of SNX27/retromer is strongly implicated in AD through increased intracellular production of β-amyloid peptides from endosomal APP breakdown (Nielsen et al., 2007; Lane et al., 2010; Fjorback et al., 2012; Willnow and Andersen, 2013; Huang et al., 2016; Milne et al., 2019). Retrograde transport of APP from endosomes to the TGN involves interaction of SorLA with retromer (Nielsen et al., 2007; Fjorback et al., 2012; Willnow and Andersen, 2013). An endosomal shunt mechanism (Nielsen et al., 2007; Huang et al., 2016) has been proposed to explain how the SNX27/SorLA interaction can shift endosomal APP trafficking toward non-amyloidogenic processing at the cell surface, but molecular details remain elusive. Neither retromer nor SNX27 have been shown to interact with APP directly, and thus SorLA has been proposed as the molecular link between SNX27/retromer function and APP processing. Therefore, it remains critical to obtain structural and molecular details surrounding the crosstalk between SorLA, SNX27, and retromer in APP trafficking and homeostasis.

### Cancers

Sorting nexin 27 is increasingly linked to cancers by mediating multiple protein–protein interactions important in trafficking, protein sorting, and membrane remodeling (Clairfeuille et al., 2016). The Cancer Genome Atlas database reveals SNX27 is highly expressed in invasive breast cancer tissue (Zhang et al., 2019; Bao et al., 2020; Sharma et al., 2020). Multiple studies have suggested how SNX27 affects tumor growth both *in vitro* and *in vivo* (Zhang et al., 2019; Sharma et al., 2020; Yang et al., 2020). SNX27 increases expression of vimentin and claudin−5 proteins, both of which promote tumor growth, and SNX27 has been proposed as a potential breast cancer biomarker (Zhang et al., 2019; Sharma et al., 2020). In breast cancer cells, SNX27 knockdown results in reduced motility, lower proliferation, less colony formation, and upregulated E−cadherin and β−catenin expression levels (Zhang et al., 2019). Additional studies using mouse models report decreased cell proliferation, tumor growth inhibition, and longer survival times (Frost et al., 2008; Pim et al., 2015). Finally, SNX27 may regulate matrix invasion by recycling specific matrix proteins, such as MT1-MMP metalloprotease, through a direct interaction (Bao et al., 2020; Sharma et al., 2020).

Understanding the underlying cell biology remains important for uncovering specific mechanisms underlying the role of SNX27 in breast cancer. SNX27 governs glucose transport by an interaction with phosphatase and tensin homolog deleted on chromosome 10 (PTEN); this prevents glucose transporter type 1 (GLUT1) accumulation at the cell surface (Steinberg et al., 2013) and suppresses cancer progression (Shinde and Maddika, 2017; Zhang et al., 2019). SNX27 affects nutrient uptake in cancer cells through recycling of different energy transport receptor proteins. Finally, SNX27 is involved in cellular uptake of specific amino acids like glutamine, as well as mTORC1 activation (Yang et al., 2018; Zhang et al., 2019), which may affect how cancer cells proliferate.

Sorting nexin 27 is also implicated in progression of acute myeloid leukemia (AML) with potential for therapeutic treatment strategies (Wermke et al., 2015). An RNA interference (RNAi) screen in primary leukemia cells linked SNX27 loss to impaired cellular growth and viability; this suggests SNX27 could be considered as a diagnostic target (Wermke et al., 2015). However, the mechanisms by which SNX27 functions in different cancers overall remain unclear. Future exploration should clarify the underlying cellular functions of SNX27/retromer in the context of specific cancer cells. Overall, SNX27 may serve as an important target based on its established roles in promoting tumorigenesis, cancer progression, and metastasis.

### Viral Pathogenesis

Retromer is targeted by numerous pathogens, including bacterial effectors and viral proteins. Viruses and their effectors have evolved many different strategies to target retromer in cells. Viral proteins can recruit retromer and cargo to replication sites to aid infection; the NS5A protein made by hepatitis C virus (HCV) interacts with VPS35 using this strategy (Yin et al., 2016; Elwell and Engel, 2018). Recruiting retromer directly to replication sites may redirect host factors that can be used to drive viral growth; one example is Tip47 (Ploen et al., 2013a, b; Vogt et al., 2013; Elwell and Engel, 2018), which has been reported to interact with CI-MPR (Diaz and Pfeffer, 1998; Orsel et al., 2000). Other viral proteins copy or mimic the motifs found in endogenous retromer cargo proteins; this likely allows them to hijack an important retrograde pathway in order to circumvent lysosomal degradation or to access genetic material in the nucleus. Both influenza virus M2 (Bhowmick et al., 2017) and HIV envelope (Env) proteins adopt this strategy (Groppelli et al., 2014). Another example is the HPV16 L2 major capsid protein that harbors multiple motifs associated with retromer; this includes ΦX (L/M), NPxY, and a non-canonical PDZ-binding motif. Together, these three sequences permit L2 to engage retromer, SNX17, and SNX27, respectively (Bergant Marusic et al., 2012; Pim et al., 2015; Popa et al., 2015; Campos, 2017); engaging all three proteins would substantially increase interaction affinity and allow the viral protein to outcompete cellular cargo. It will be interesting to determine biochemically whether most viral proteins directly bind retromer or one of the sorting nexins now proposed as cargo adaptors.

Some viral effectors instead change the activity or localization of retromer. One example is tyrosine kinase-interacting protein (Tip) from Herpesvirus saimiri; this protein redistributes VPS35 from early endosomal membranes to lysosomes (Kingston et al., 2011). The Tip protein is not required for replication, but retromer inhibition by Tip may contribute to transformation observed in T cells (Duboise et al., 1998). Human papilloma virus (HPV) E6 protein interacts with the SNX27 PDZ domain and affects GLUT1 trafficking; this interaction drives substantially increased glucose uptake and has been proposed to explain HPV malignancy (Ganti et al., 2016). The non-essential Vaccinia virus K7 protein interacts with both VPS26 and VPS35 (Li et al., 2017), and this interaction has been suggested to affect virus transport or uncoating (Benfield et al., 2013).

Recently, multiple groups used orthogonal methods including CRISPR knockout, RNA interference, proteomics, and small-molecule inhibitors, to show retromer and SNX27 may be involved in SARS-CoV-2 viral life cycle and infection (Daniloski et al., 2020; Zhu et al., 2020). One group identified host genes required for SARS-CoV-2 infection in a human A549 (lung adenocarcinoma) cell line that overexpresses the ACE2 receptor (Papa et al., 2020). Another group (Cattin-Ortolá et al., 2020) identified SNX27/retromer and other trafficking coat complexes as important host factors that influences spike (S) protein sorting in cells. Overall, understanding the fundamental trafficking pathways and mechanisms that govern SNX27/retromer assembly and regulation are likely to provide key insights into how pathogens hijack host cells.

## Perspective

Many important studies have now linked SNX27 to physiology and disease. It is now vital for the field to move toward integrating structural and biochemical data with experiments in model systems to understand the molecular role of SNX27 in cellular pathways linked to human disease. The field needs additional biochemical and structural information to judge the suitability of SNX27 as a viable therapeutic target. Structural data have demonstrated how SNX27 engages PDZ motifs, and indirect evidence from a related protein (SNX17) suggests how the FERM domain likely binds NPxY motifs. It is difficult to envision targeting either of these binding pockets with a small molecule, because SNX27 sorts many different important cellular cargoes. Broad disruption of cargo binding, especially for diseases requiring long term intervention, would likely have undesirable physiological effects. Such an approach may have merit for shorter treatments, such as inhibiting pathogen binding. Furthermore, the field needs to determine how SNX27 engages cargos in the context of binding both retromer and membranes. This would allow us to understand what conformation SNX27 adopts when bound to retromer, cargo, and phospholipids. Such studies would also reveal whether (or not) SNX27/retromer possesses interfaces distinct from those found in SNX-BAR/retromer or SNX3/retromer coats. If yes, might specific SNX27/retromer interfaces be stabilized or destabilized by small molecules, depending on disease? These are exciting and important questions to explore in the coming years.

## Author Contributions

MC and AK wrote early drafts. MC made figures. LJ conceived the review, assisted in writing structural sections, and undertook editing with input from all authors. All authors contributed to the article and approved the submitted version.

## Conflict of Interest

The authors declare that the research was conducted in the absence of any commercial or financial relationships that could be construed as a potential conflict of interest.
